# Association between single-nucleotide polymorphisms of *NKX2.5* and congenital heart disease in Chinese population: A meta-analysis

**DOI:** 10.1515/biol-2022-0058

**Published:** 2022-05-12

**Authors:** Huan Chen, Tianjiao Li, Yuqing Wu, Xi Wang, Mingyuan Wang, Xin Wang, Xiaoling Fang

**Affiliations:** Department of Obstetrics and Gynecology, The Second Xiangya Hospital of Central South University, Changsha, Hunan, China; Department of Pathophysiology, Xiangya Medical College, Central South University, Changsha, Hunan, China

**Keywords:** congenital heart disease, *NKX2.5*, SNP, meta-analysis, case–control study, CRD42020185109

## Abstract

*NKX2.5* is a transcription factor that plays a key role in cardiovascular growth and development. Several independent studies have been previously conducted to investigate the association between the single-nucleotide polymorphism (SNP) 606G >C (rs3729753) in the coding region of *NKX2.5* and congenital heart disease (CHD). However, the results of these studies have been inconsistent. Therefore, the present study aimed to reveal the relationship between *NKX2.5* SNP 606G >C and the risk of CHD as possible in the Chinese population through meta-analysis. After retrieving related articles in PubMed, MEDLINE, EMBASE, Web of science, Cochrane, China National Knowledge Infrastructure, Wanfang DATA, and VIP database until August 2021, a total of eight studies were included in the present meta-analysis. The qualified research data were then merged into allele, dominant, recessive, heterozygous, homozygous, and additive models. Overall results of the current meta-analysis showed that 606G >C was not associated with CHD of the Chinese population in any model. In addition, subgroup analysis based on CHD type gave the same negative result. Results of sensitivity analysis showed that there was no significant correlation after the deletion of each study. Furthermore, it was noted that the results were negative and the heterogeneity was not significant. In conclusion, it was evident that *NKX2-5* SNP 606G >C may not lead to the risk of CHD in Chinese population.

## 1 Introduction

Congenital heart disease (CHD) refers to a severe structural abnormality of the heart or intrathoracic blood vessels that occurs at birth [1]. Common types of CHD include atrial septal defect (ASD), ventricular septal defect (VSD), and patent ductus arteriosus. CHD is one of the most common types of deformities in humans [2] and also among the leading cause of neonatal death [3]. Globally, the incidence of CHD is estimated to be 9.1 per 1,000 live births [4], whereas in China, between 800,000 and 1.2 million children are born with birth defects annually, including about 220,000 cases of CHD [5].

Presently, the diagnosis of congenital fetal abnormalities mainly depends on prenatal ultrasound screening and laboratory tests [6], whereas computed tomography, magnetic resonance imaging, and echocardiography are also common imaging methods used to diagnose CHD [7]. Laboratory methods for prenatal diagnosis include cytogenetics and the biochemical examination of amniotic fluid among others. The gold standard for invasive prenatal diagnosis is classic cytogenetic analysis [8]. With the current advancement of medical technology, the survival rate of patients with CHD has also significantly increased [9]. CHD is a multifactorial disease related to both genetic and environmental factors [10]. Furthermore, the causes of birth defects have also been widely discussed, but have not yet been fully elucidated [11]. By identifying the source of the defect, the defect rate can also be reduced or eliminated [12].

The human homeobox gene *NKX2.5* is the earliest discovered gene related to human heart development. It is located at the far end of chromosome 5, the 5q34–q35 region. The human homeobox gene *NKX2.5* is abundantly expressed in the human fetal heart, which suggests that it plays a key role in human heart development [13]. It has been reported that deletion of *NKX2.5* can cause abnormalities in the morphology of the embryonic heart, growth retardation, and embryo death in mice [14], as well as malformations in *Xenopus laevis* hearts [15]. Therefore, these findings further confirmed the important role of *NKX2.5* in cardiac development.

Several studies have found that the *NKX2.5* mutation is associated with a variety of human heart malformations [16,17,18,19,20,21,22,23,24,25]. A total of about 105 *NKX2.5* mutations has been discovered that include synonymous mutations, missense mutations, insertion mutations, and deletion mutations. The evaluation of the *NKX2.5* mutation can not only provide a basis for early diagnosis of CHD, but also identify the family members who may be at risk of contracting the heart disease [26]. The *NKX2.5* single-nucleotide polymorphism (SNP) 63A >G (rs2277923, Glu21Glu) and 606G >C (rs3729753, Leu202Leu) are the two SNPs that have been studied more frequently in CHD.

The latest meta-analysis has found that there was no correlation between the *NKX2.5* gene 63A >G (rs2277923, Glu21Glu) polymorphism and CHD susceptibility in Chinese and non-Chinese populations [27]. Furthermore, it has been reported that 606G >C (rs3729753, Leu202Leu) is a synonymous mutation of *NKX2.5* and a large number of independent studies have also explored its relationship with CHD. However, the results are still controversial [28,29,30,31,32,33,34,35,36,37,38,39,40,41,42,43,44,45]. Therefore, the current meta-analysis was conducted because the statistical power of individual research was not enough to better assess the relationship between the *NKX2.5* gene 606G >C polymorphism and the risk of Chinese CHD.

## 2 Methods and materials

### 2.1 Literature search strategy

The following keywords were used for literature search in the current study: “Homeobox Protein Nkx 2.5,” “Nkx-2.5, Homeobox Protein,” “NK2 Homeobox 5 Protein,” “Homeobox Transcription Factor Csx-Nkx2-5,” “Homeobox Transcription Factor Csx Nkx2 5,” “Homeobox Protein Csx-Nkx2.5,” “Csx-Nkx2.5, Homeobox Protein,” “Homeobox Protein Csx Nkx2.5,” “Cardiac-Specific Homeobox Protein,” “Cardiac Specific Homeobox Protein,” “Homeobox Protein, Cardiac-Specific,” “Transcription Factor Nkx-2.5,” “Nkx-2.5, Transcription Factor,” “Transcription Factor Nkx 2.5,” “Nucleotide Polymorphism, Single,” “Nucleotide Polymorphisms, Single,” “Polymorphisms, Single Nucleotide,” “Single Nucleotide Polymorphisms,” “SNPs,” “Single Nucleotide Polymorphism” to carry out an unlimited search of electronic databases of PubMed, MEDLINE, EMBASE, Web of science, Cochrane, China National Knowledge Infrastructure, Wanfang DATA, and VIP database.

The initial search was conducted on September 1, 2021, and the relevant articles were identified as well as published as of August 31, 2021. The meta-analysis was conducted and the results reported based on the preferred reporting items stated by System Review and Meta-Analysis (PRISMA).

The inclusion criteria for the present study were formulated before the literature search. Therefore, eligible studies met the following conditions: (1) Articles that have been published electronically; (2) Case–control studies of unrelated CHD patients and healthy controls; (3) Evaluation of the correlation between *NKX2.5* 606G >C polymorphism (rs3729753) and the risk of CHD; (4) Provided sufficient genotype data to calculate odds ratios (ORs), corresponding 95% confidence intervals (CIs) and could be able to establish various genetic models; (5) All texts are available in English or Chinese; (6) Histological or pathological confirmation of non-syndromic CHD; all clinical types, such as ASD and VSD, were included in this meta-analysis. Pedigree studies, case reports, case series, reviews, editorials, the meta-analysis, animal studies, expert opinions, and studies that consider CHD as part of any known genetic disease or multiple congenital abnormalities syndrome were also excluded in the present meta-analysis.

### 2.2 Data extraction

The two reviewers independently extracted the following information from the included studies: first author, year of publication, country, type of study design, types of CHD, genotype and gene frequency in patients with CHD as well as control group, and whether the *NKX2.5* gene polymorphism was in accordance with Hardy–Weinberg equilibrium (HWE) in the control group.

The Newcastle-Ottawa Quality Assessment Scale, which is a classic assessment tool, was used to evaluate the quality of non-randomized studies from the perspectives of selection, comparability, exposure, and evaluate the effectiveness of all case–control studies. Two reviewers (HC and XW) independently performed data extraction and quality evaluation. Furthermore, the reviewer would write to the authors to obtain additional information or original data when necessary.

### 2.3 Statistical analysis

All data were analyzed using Stata software version 16.0 (Stata Corp LP, TX, USA). The statistical significance of difference was set at *P*-value <0.05. Furthermore, Chi-square test was used to calculate the *P* value of the control group HWE. The association between SNP 606G >C and susceptibility to CHD was estimated using combined ORs and 95% CIs under different genetic models. Cochran’s Q statistical test was also conducted to assess heterogeneity between various studies. If the probability value (*P*-value) was less than 0.1 or *I* ^2^ is greater than 50%, the random effect model was employed because of the significant heterogeneity; otherwise, the fixed-effect model was applied. Consequently, the subgroup analysis based on the type of CHD was then performed. Sensitivity analysis was performed to assess the impact of each individual study on the overall estimate, whereas Begg’s and Egger’s tests were finally used to assess potential publication bias.

## 3 Results

### 3.1 Literature inclusion

**Figure 1:**
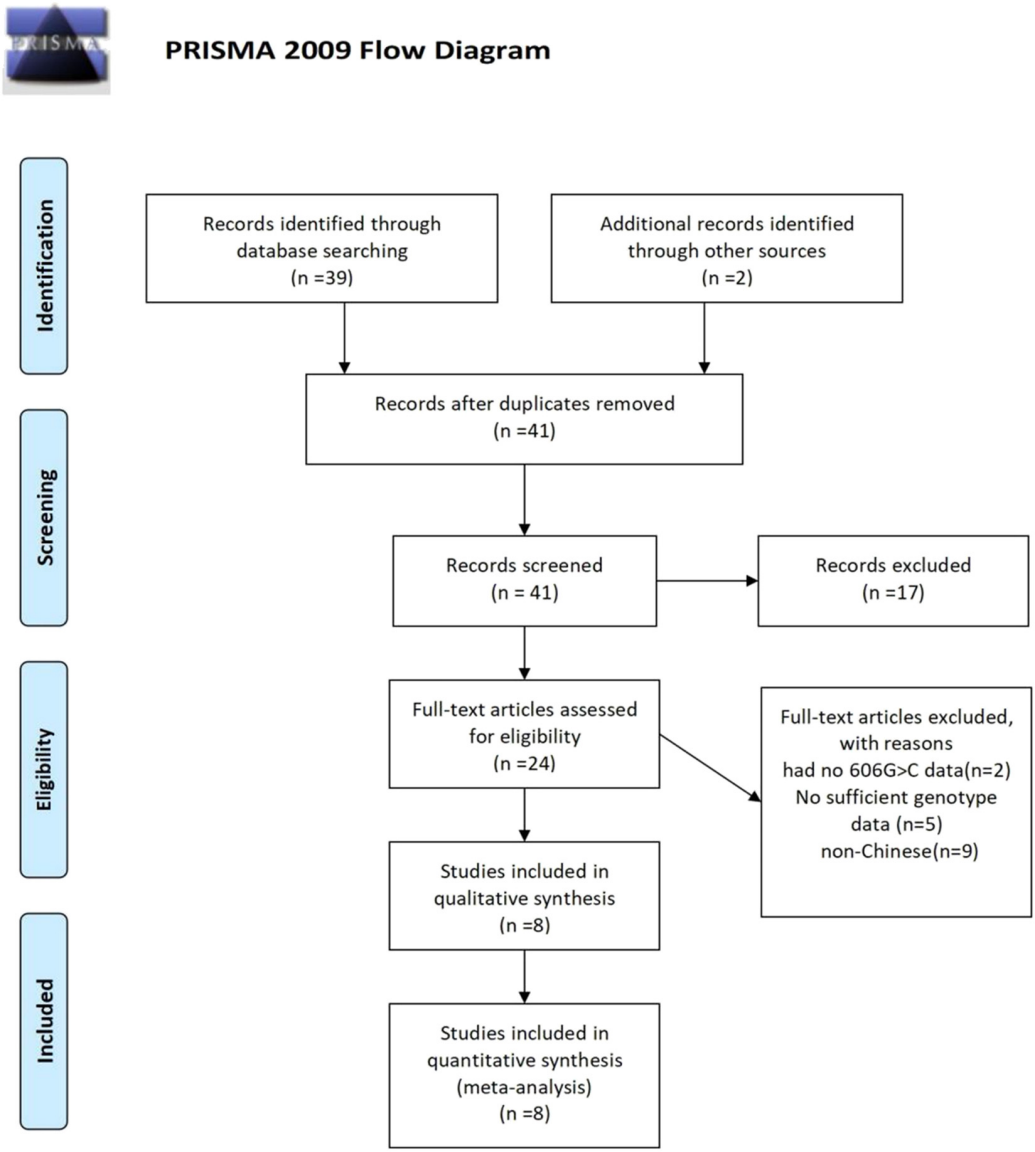
Flowchart of this study [46].

The document retrieval and selection process was as shown in the flow chart in Figure 1. A total of 53 documents were found in the initial search. Furthermore, irrelevant or duplicated articles were excluded after reading the title and abstract, whereas 24 articles were selected for further evaluation. In addition, 16 more publications were excluded after review of the full texts because of the following reasons: Two studies had no 606G >C data, nine studies were conducted among non-Chinese, eight of these studies were unable to provide complete data to build various genetic models and complete allele data were also not obtained in the other five studies based on Chinese population.

Finally, a total of eight studies including four published in English and four in Chinese language (1,361 cases and 2,030 controls) were included in the present meta-analysis. The HWE test of each control group included in the study was consistent with HWE. Among these studies, two studies explored only one type of CHD, three studies explored only ASD and VSD, and all of the studies used the same population as a control group. The remaining studies involved multiple types of CHD, and characteristics of the included studies are shown in Table 1.

**Table 1:** Characteristics of the included documents

First author	Year	Country	CHD case			Control			Phenotype
				Genotypes	Alleles		Genotypes	Alleles	
			*n*	GG/GC/CC	G/C (%)	*n*	GG/GC/CC	G/C (%)	
Yin, J.	2019	China	98	92/6/0	96.9/3.1	200	189/11/0	97.3/2.7	Multiple
Cao, Y.^a^	2016	China	107	101/6/0	97.2/2.8	487	465/22/0	97.7/2.3	ASD
Cao, Y.^a^	2016	China	385	367/18/0	95.3/4.7	487	465/22/0	97.7/2.3	VSD
Zhang, W.	2016	China	120	116/4/0	98.3/1.7	120	117/3/0	98.7/1.3	ASD
Cao, Y.	2015	China	70	68/2/0	98.6/1.4	136	131/5/0	98.2/1.8	Multiple
Tang, J.^a^	2015	China	50	48/2/0	98/2	50	47/3/0	97/3	VSD
Tang, J.^a^	2015	China	51	49/2/0	98/2	50	47/3/0	97/3	ASD
Zhao^a^	2014	China	40	37/3/0	96.2/3.8	50	45/5/0	95/5	ASD
Zhao^a^	2014	China	50	47/3/0	96.9/3.1	50	45/5/0	95/5	VSD
Zhang, W.	2009	China	230	219/11/0	97.6/2.4	130	118/12/0	97/3	Multiple
Liu, X. Y.	2009	China	160	145/15/0	95.3/4.7	200	191/9/0	97.8/2.2	VSD

a: These three studies shared identical control subjects.

### 3.2 Overall meta-analysis

**Figure 2:**
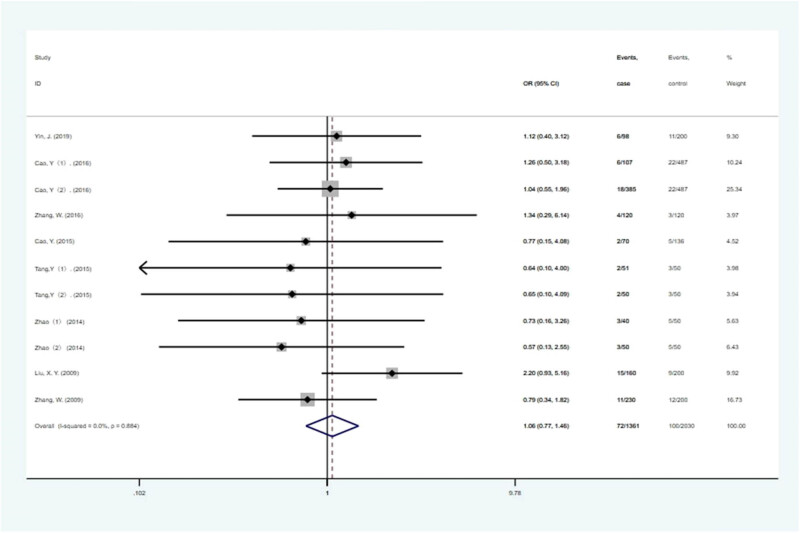
Forest plot on association between NKX2-5 606G >C polymorphism and CHD risk (heterozygous gene model). *NKX2.5* 606G >C SNP was not significantly related to the occurrence of CHD.

Combined results of the 606G >C dominant model in the current study are shown in Figure 2. Furthermore, a fixed-effects model was used in the present study because heterogeneity was not significant (*P* = 0.884). In the heterozygous gene, allele gene, dominant gene, and additive gene models, it was found that *NKX2.5* 606G >C SNP was not significantly related to the occurrence of CHD. Results of various models are also indicated in Table 2. Furthermore, the recessive gene and homozygous gene models could not be analyzed in the current study because the CC data of 606G >C was zero.

**Table 2:** Overall analysis results of various models

Model	OR	95% CI	*P*	*I* ^2^ (%)
Heterozygote	1.062	0.772–1.461	0.884	0.0
Allele	1.061	0.774–1.452	0.896	0.0
Dominant	1.062	0.772–1.461	0.884	0.0
Additive	0.941	0.684–1.295	0.884	0.0

### 3.3 Subgroup analysis

**Figure 3:**
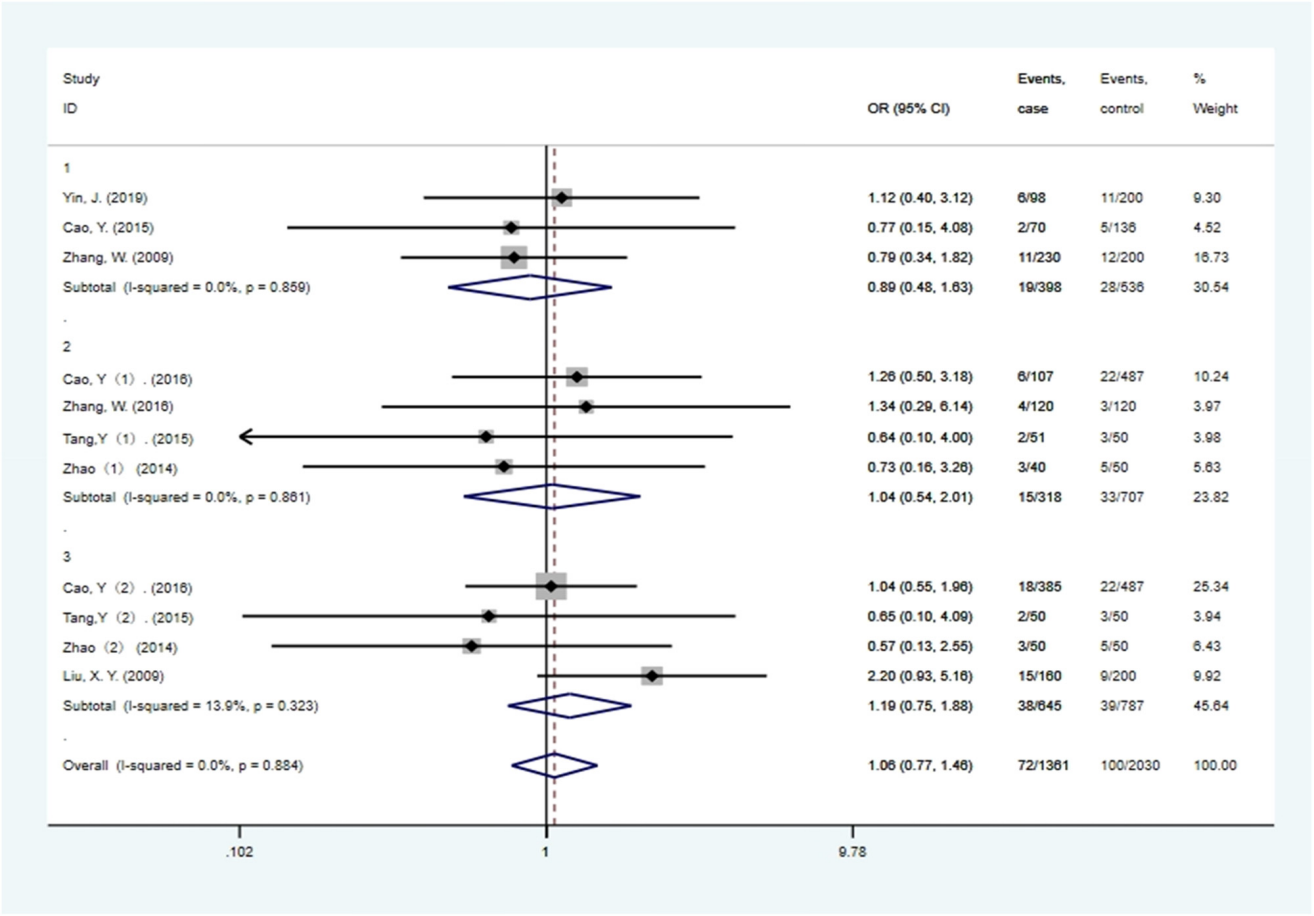
The subgroup forest plot on association between NKX2-5 606G >C polymorphism and CHD risk (heterozygous gene model). There is no significant correlation between *NKX2.5* 606G >C polymorphism and CHD.

A subgroup analysis of existing data was performed in the present study for the *NKX2.5* 606G >C polymorphism and CHD risk based on the type of CHD in the study population. In multiple, VSD, and ASD subgroups (Figure 3), the current study failed to find any significant correlation between *NKX2.5* 606G >C polymorphism and CHD in any of the studied models (Table 3).

**Table 3:** Subgroup analysis results of various models

	Heterozygote					Allele				
	OR	CI	*P*	*I* ^2^	Ph	OR	CI	*P*	*I* ^2^	Ph
Multiple	0.886	0.483–1.626	0.696	0.0%	0.859	0.889	0.488–1.618	0.700	0.0%	0.862
ASD	1.043	0.542–2.009	0.899	0.0%	0.861	1.045	0.547–1.994	0.894	0.0%	0.871
VSD	1.190	0.753–1.883	0.323	13.9%	0.323	1.183	0.753–1.859	0.466	11.4%	0.336

	Dominant					Additive				
Multiple	0.886	0.483–1.626	0.696	0.0%	0.859	1.129	0.615–2.072	0.696	0.0%	0.859
ASD	1.043	0.542–2.009	0.899	0.0%	0.861	0.956	0.496–1.841	0.893	0.0%	0.859
VSD	1.190	0.753–1.883	0.323	13.9%	0.323	0.841	0.532–1.331	0.319	14.6%	0.46

### 3.4 Sensitivity analysis

Although results of the present study showed that there was no significant heterogeneity between studies, a sensitivity analysis was still conducted to assess the impact of each study on the overall estimate. Sensitivity analysis was performed by removing one individual study at each time. For the subgroup 606G >C polymorphism, it was also found that deleting any study did not affect the overall results. Furthermore, relatively robust results were found in the sensitivity analysis of the current study.

### 3.5 Publication bias

**Figure 4:**
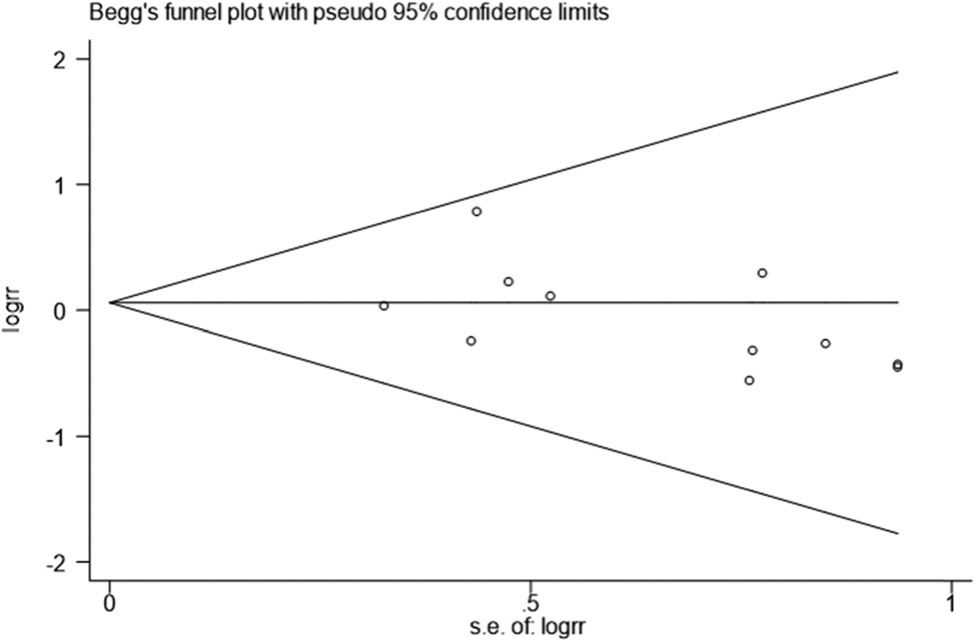
Begg’s funnel plot.

**Figure 5:**
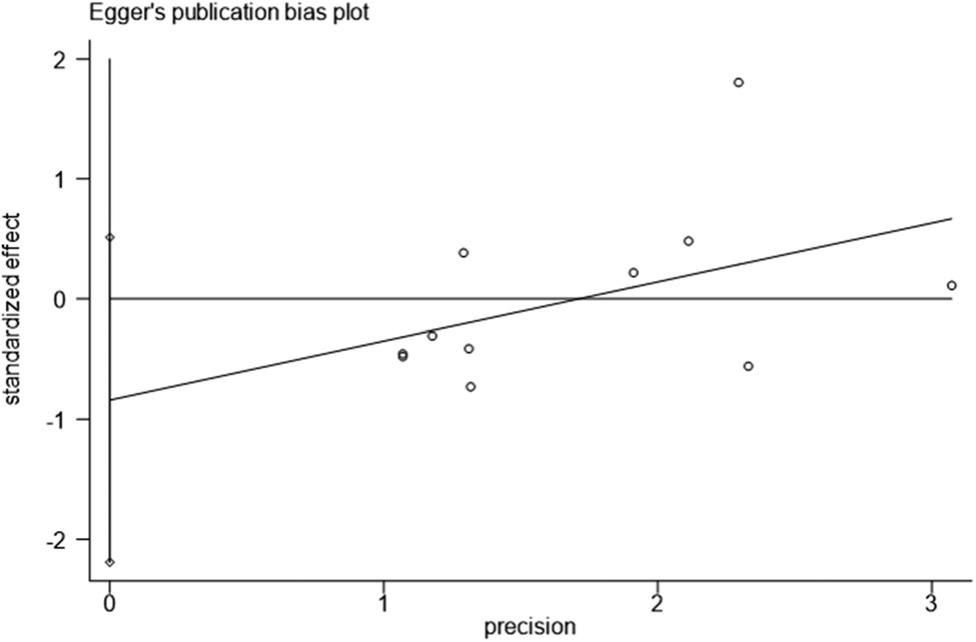
Egger’s publication bias plot.

Results of Begg’s and Egger’s tests in the current study which were used to detect publication bias found that there was no publication bias for *NKX2.5*, 606G >C (Pr > |*z*| = 0.350) (Figures 4 and 5).

## 4 Discussion

In most societies, the most common deaths are related to heart disease and CHD accounts for a large proportion, especially among infants as well as children [47]. Furthermore, it has been reported that genetic factors play an important role in development of CHD [48]. Prenatal diagnosis is an important means for prevention of CHD [49]. Moreover, accurate genetic counseling and prenatal examination can be provided through genetic diagnosis [50] to reduce the rate of birth defects.

The transcription factors *NKX2.5*, *GATA 4*, *Myocardin*, and *Tbx20* play a vital role in both cardiac morphogenesis and differentiation. During development, the expression of *NKX2.5* precedes the expression of other known heart-specific genes [51]. It was found that mice with the targeted disruption of the *NKX2.5* gene showed cardiac developmental arrest during circulatory stage and died when still in the uterus. Its gene mutation can lead to the key determinant of cardiac morphology – circular morphogenesis does not start [14]. Furthermore, it was reported that mutation of *NKX2.5* homeodomain seriously reduced the DNA binding activity, with little or no transcription activation function [52] and hence resulted in ASD, Junctional ASDs, other ventricular septal abnormalities, and mild conduction defects [53]. In embryonic, it was found that neonatal, and adult mice models, progressive and severe cardiac conduction defects as well as heart failure occurred [54,55].

To date, there has not been any functional research on 606G >C polymorphism (Leu202Leu). It locates in exon 2 of *NKX2.5* gene. Although this SNP does not change the amino acid sequence and structure of the *NKX2.5* domain, several studies have found that this polymorphism has no significant difference between sporadic CHD and healthy controls. However, some studies have also reached the opposite conclusion, which suggests that this polymorphism may cause CHD [28,30]. Notably, it was found that none of the studied articles provided complete genotype and allele numbers and thus it was not included in the study. Furthermore, both were published in 2018 and beyond. Therefore, the current meta-analysis was conducted to resolve the conflict and obtain a more conclusive result. Results of the current study suggested that the *NKX2.5* 606G >C polymorphism may not be related to the risk of CHD and this finding was in agreement with the results of the previous meta-analysis [56,57,58,59].

Although the results of the current study were consistent with the results of the previous meta-analysis, the results of the current study were more reliable and had some strengths different from other previous studies. First, considering our findings that *NKX2.5* 606G >C polymorphism was based on more qualified and relatively high-quality studies, the sample size of this analysis was significantly larger than the size in the previous studies (Table 4); second, to the best of our knowledge, the present meta-analysis analyzed six genetic models (heterozygous, allele, dominant, additive, recessive, and homozygous), for the time which gave a broader picture of the *NKX2.5* genotype involved, rather than just the role of alleles; third, the current study further confirmed that polymorphism has no obvious relationship with CHD.

**Table 4:** Characteristics of previous meta-studies

Author	Year	Case number	Control number	Result
Wang, Z.	2013	748	630	Negative
Xie, X.	2016	1330	1167	Negative
Chen, L. T.	2018	978	937	Negative
Gonzalez-Castro	2021	890	953	Negative

In previous meta-analyses, scholars also analyzed the association of the other two *NKX2.5* SNPs (rs2277923 and rs703752) with CHD, with varied results. The etiology of CHD is complex and is associated with environmental and genetic factors. Different geographic regions, living environments, and dietary structures can lead to differences in the frequency of gene polymorphism distribution and hence genetic susceptibility, whereas the lack of data on environmental effects on genes and gene interactions limits the analysis of interactions. The literature included in each meta-analysis are different and these may have contributed to the inconsistent results.

This study had some limitations: first, the number of studies that studied the relationship between *NKX2.5* gene polymorphisms and risk of CHD was limited, especially the number of studies that include genotype and allele results and only some studies the data could be integrated. Second, this meta-analysis was only based on the Chinese population. There was only one non-Chinese study that could provide enough data to establish various genetic models and hence the role of this polymorphism in other ethnic groups could not be known. Third, all the studies included in the present meta-analysis were published in English or Chinese and hence some qualified articles in published in other languages might have been missed out. Fourth, the sample size was relatively small. In addition, all the included studies were in a retrospective study design. Therefore, there is need for other large-scale and well-designed studies to further confirm results of the present study. Fifth, it was impossible to obtain some basic data for comparison, such as the ratio of men to women, and age, among others which prevents further analysis.

## 5 Conclusion

In conclusion, the current meta-analysis shows that *NKX2.5* 606G >C polymorphism has no significant correlation with pathogenesis of Chinese CHD. However, considering that the current results are based on a limited number of case–control studies and are limited to studies in Chinese populations, there is need to draw more credible conclusions in a multicenter, larger sample, high-quality case–control study. Furthermore, homeobox transcription factor *NKX2.5* is a key regulator of cardiac gene expression, cardiac development, and it is also the most studied cardiac development gene. Therefore, although results of the current meta-analysis were negative, there is need for more research on the role of this gene and its polymorphism in the pathogenesis of CHD.
